# Allele-specific alternative splicing of *Drosophila Ribosomal protein S21* suppresses a lethal mutation in the *Phosphorylated adaptor for RNA export* (*Phax*) gene

**DOI:** 10.1093/g3journal/jkac195

**Published:** 2022-08-03

**Authors:** Eric L Garcia

**Affiliations:** Department of Microbiology, Immunology and Molecular Genetics, University of Kentucky College of Medicine, Lexington, KY 40536, USA; Department of Biology, University of Kentucky, Lexington, KY 40506, USA; Integrative Program for Biological and Genome Sciences, University of North Carolina at Chapel Hill, Chapel Hill, NC 27599, USA

**Keywords:** phosphorylated adaptor for RNA export protein, Phax, ribosome, ribosomal protein, ribosomal protein S21, RpS21, alternative splicing, alternative polyadenylation, mRNA processing, snRNP biogenesis, Spinal Muscular Atrophy, *oho23B*, splicing factor, *cis*-element, exonic splicing enhancer, B52, survival motor neuron

## Abstract

Genetic disruptions to the biogenesis of spliceosomal small-nuclear ribonucleoproteins in *Drosophila* cause wide-spread alternative splicing changes, including changes to the splicing of pre-mRNA for Ribosomal protein S21 (RpS21). Using a transposon mutant for the *Phosphorylated adaptor for RNA export* (*Phax*) gene, we demonstrate that changes in the splicing of *RpS21* transcripts have a strong influence on the developmental progression of *Phax^SH/SH^* mutants. Different alleles of the *Drosophila RpS21* gene are circulating in common laboratory strains and cell lines. These alleles exhibit differences in *RpS21* intron retention and splicing efficiency. Differences in the splicing of *RpS21* transcripts account for prior conflicting observations of the phenotypic severity of *Phax^SH/SH^* mutant stocks. The alleles uncover a strong splicing enhancer in *RpS21* transcripts that can fully suppress the larval lethality and partially suppress the pupal lethality exhibited by *Phax^SH/SH^* mutant lines. In the absence of the splicing enhancer, the splicing of *RpS21* transcripts can be modulated in *trans* by the SR-rich B52 splicing factor. As *Phax^SH/SH^* mutants exhibit wide-spread splicing changes in transcripts for other genes, findings here establish the importance of a single alternative splicing event, *RpS21* splicing or intron retention, to the developmental progression of *Drosophila*.

## Introduction

Depletion of core splicing machinery leads to specific, nonrandom changes in alternative splicing and gene expression, including changes to ribosomal protein (RP) coding genes (Pleiss *et al.* 2007; Saltzman *et al.* 2011; Garcia *et al.* 2016). Conversely, targeted inhibition of the transcription of highly expressed RP coding genes liberates the “hungry” spliceosome and results in distinct changes to alternative splicing (Munding *et al.* 2013; Talkish *et al.* 2019). Similarly, disease-associated mutations in RP coding genes and corresponding reductions in ribosomes cause specific changes in gene expression and the translation of select transcripts (Horos *et al.* 2012; Khajuria *et al.* 2018). The specificity of responses to different perturbations in the overall gene expression program supports an interconnected regulatory network for the biogenesis and homeostatic regulation of the spliceosome and the ribosome that intersects in the RNA processing of *RP* pre-mRNA.

Our prior transcriptomic profile of small-nuclear ribonucleoprotein (snRNP) biogenesis mutants in *Drosophila* identified wide-spread alternative splicing changes in *Phosphorylated adaptor for RNA export* (*Phax*) mutants and *Survival motor neuron* (*Smn*) mutants (Garcia *et al.* 2016). Phax functions as a small-nuclear RNA (snRNA)-specific adapter to export Sm-class snRNAs from the nucleus, and Smn functions to assemble exported snRNAs into snRNPs in the cytoplasm (Ohno *et al.* 2000; Ohno 2012; Matera and Wang 2014). Full loss of fly Smn function results in a recessive larval lethality in *Drosophila* (Chan *et al.* 2003; Rajendra *et al.* 2007; Chang *et al.* 2008). In contrast, lethality can be genetically separated from a disruptive P element insertion in the *Drosophila Phax* gene, an insertion previously classified as lethal (Oh *et al.* 2003; Kahsai *et al.* 2016). Our lab and others have observed adult flies that are homozygous for this P element insertion in the *Phax* gene (Kahsai *et al.* 2016). Nevertheless, both *Phax* and *Smn* mutants exhibit lower steady-state levels of Sm-class snRNAs and an overlapping set of alternative splicing changes that includes changes in *RP* pre-mRNA, including a large change in the alternative splicing of pre-mRNA for Ribosomal protein S21 (RpS21) (Garcia *et al.* 2016).

RpS21 is component of the small subunit of the ribosome with functions in translation initiation and ribosome biogenesis (Török *et al.* 1999; Sato *et al.* 2003; Tabb-Massey *et al.* 2003; Black and Johnson 2022). In *Drosophila*, haploinsufficient *RpS21* mutants exhibit the classic *Minute* phenotype of short and thin bristles, prolonged development, and impaired viability (Török *et al.* 1999; Marygold *et al.* 2007). Homozygous *RpS21* mutants display an overgrowth of hematopoietic organs like the overgrowth observed with fly *stubarista* (*sta/RpSA/P40*) and *Ribosomal protein S6* (*RpS6*) mutants (Watson *et al.* 1992; Stewart and Denell 1993; Török *et al.* 1999). Reduced levels of the core snRNP protein SmD3 have also been linked to a similar extension of development and concomitant overgrowth of hematopoietic tissues (Schenkel *et al.* 2002). In the context of our identification of large changes in *RpS21* alternative splicing in other snRNP biogenesis mutants, the similarity in phenotypes between *RpS21* and *SmD3* mutants suggests that changes in *RpS21* alternative splicing may impact developmental progression and organismal viability.

Here, we identify alternative alleles of *RpS21* that encode transcripts with dramatic differences in alternative splicing. The different alleles uncover sequences in *RpS21* exon 4 that function to enhance the splicing of exon 3 to exon 4. Remarkably, the *RpS21* allele with the splicing enhancer sequences was capable of suppressing the lethality of a *Phax* P element insertion mutant. Hence, findings here reveal a discrete genetic background difference that can account for prior conflicting observations of the viability of different fly stocks from this *Phax^SH/SH^* mutant line. Importantly, in the background of wide-spread alternative splicing changes, restoration of a single splicing event in *RpS21* was ultimately able to rescue the developmental progression of homozygous *Phax^SH/SH^* mutant flies.

## Materials and methods

### Fly strains and husbandry

Stocks were maintained on cornmeal and agar at room temperature (25 ± 1°C) in half-pint bottles. For crosses, sorting and the isolation of larvae, stocks were cultured on molasses and agar at room temperature (23 ± 3°C) unless indicated otherwise. Stocks for wild-type *Ore-R* (Oregon-R-modENCODE) and the reference genome strain *iso-1* (y[1]; Gr22b[1] Gr22d[1] cn[1] CG33964[R4.2] bw[1] speck[1]; MstProx[1] GstD5[1] Rh6[1]) were obtained directly from the Bloomington *Drosophila* Stock Center (BDSC). Stocks for *Canton-S* (*Can-S*) and *w^1118^* were obtained from D.A. Harrison. Recombination of the *UAS:Phax-mVenus* transgene into the mutant (P{lacW} Phax^SH0641^) (Oh *et al.* 2003) was previously described (Garcia *et al.* 2016). Here, *Phax^SH/SH^* refers to homozygous lines with the P element insertion in the *Phax* gene (P{lacW} Phax^SH0641^). The *armadillo* (*arm*) promoter-GAL4 driver P{GAL4-arm. S}11 (Sanson *et al.* 1996) was maintained in the *Phax^SH/SH^* background.

To generate *Phax^SH/SH^* mutant flies that were heterozygous and homozygous for the different *RpS21* alleles, we crossed virgin female flies with a second chromosome balancer (*Cyo*; *Actin-GFP*), the *RpS21^S^* short allele, and the P element insertion in the *Phax* gene to *Canton-S* wild-type male flies, homozygous for the *RpS21^L^* long allele, described below. Meiotic recombination was allowed to take place in noncurly virgin female progeny from this initial cross. These virgin females were crossed to curly male progeny with the *RpS21^L^* allele and the *Cyo; Actin-GFP* balancer. Progeny from this cross were genotyped to select a balanced recombinant with the *RpS21^L^* long allele and the P element in the *Phax* gene, using a proteinase K (New England Biolabs Inc) method for the nonlethal isolation of DNA from *Drosophila* wings (Carvalho *et al.* 2009). Separate crosses were set up with balanced flies, containing the P element insertion in the *Phax* gene and different combinations of the *RpS21^L^* long and *RpS21^S^* short allele. Through genotyping of unbalanced larvae, we isolated 3 separate stocks for each of the following genotypes: *Phax^SH/SH^*, *RpS21^L/L^*; *Phax^SH/SH^*, *RpS21^S/L^*; and *Phax^SH/SH^*, *RpS21^S/S^*.

For viability experiments, larvae were sorted on molasses-agar plates and transferred to cornmeal-agar vials. We sorted an average number (*n*) of 76 larvae for each replicate of the 23°C *GAL4:UAS* viability experiment, 100 larvae for the 27°C *GAL4:UAS* study, and 30 larvae for the viability study with homozygous or heterozygous *RpS21* alleles in the *Phax^SH/SH^* mutant background. A Student’s *t*-test was used to calculate *P*-values.

## Genotyping

DNA from whole animals or cells was isolated for genotyping with a Quick-DNA Tissue/Insect Miniprep Kit (Zymo Research) and a 4-place Mini Bead Mill Homogenizer (VWR) according to the manufacturer’s protocol. Oligonucleotides used for PCR with Apex Taq Red (Genesee Sci) of the long allele or the short allele are listed in Supplementary Table 1. Gene-flanking oligonucleotides were used to clone the *RpS21* gene from *Oregon-R* flies, S2-DRSC cells, and Kc167 cells into the *HindIII* and *BamHI* sites of pBlueScript II SK+ (Agilent) for sequencing with M13 forward and reverse primers. To genotype the *Phax^SH/SH^* mutant, we used 2 sets of oligonucleotides. We used primers complementary to the *Phax* gene that flanked the P element insertion, and we used a P element out primer, an M13 sequencing primer, with a primer complementary to the 3′-end of the *Phax* gene. This oligonucleotide set confirmed the inverse directionality of the P element in relationship to the forward direction of the *Phax* gene. For primer design, gene and intergenic sequences were derived from Flybase and compatible with the current release FB2022_02 http://flybase.org/ (Larkin *et al.* 2021).

### Splicing mini-gene reporter design

Different wild-type and mutant *RpS21* gene sequences, surrounding intron 3, were subcloned into the *KpnI* and *BamHI* sites in *pAc5.1B-EGFP* (Addgene plasmid # 21181; Elisa Izaurralde depositor). Wild-type and mutant gene sequences were generated as double-stranded DNA gene fragments (IDT gBlocks). Sequences for the minimal splicing reporter corresponded to the *RpS21* gene from nucleotide 400 to nucleotide 611, based on the *iso-1* reference sequence. These *RpS21* gene sequences spanned the third intron from exon 3, 104 nucleotides upstream of the 5′-splice site, to immediately downstream of the AATAAA polyadenylation signal in exon 4, 50 nucleotides downstream of the intron 3 3′-splice site.

Cellfectin II Reagent (Gibco) was used for transfections of S2-DRSC according to the manufacturer’s protocol. RNA was isolated on day 3 post transfection. RT-PCR was performed as indicated below with mini-gene specific primers (Supplementary Table 1). PCR products were separated on an agarose gel, stained with GelRed (Biotium), and imaged on a Gel Doc Ez (*BIO-RAD*). Bands were quantified with Image Lab 6.0.1 software (*BIO-RAD*). Percent Spliced In (PSI) was quantified as the percentage of spliced over total transcripts, and *P*-values were determined with a Student’s *t*-test.

### Cell culture and RNA interference

*Drosophila* S2-DRSC (DGRC Stock 181; https://dgrc.bio.indiana.edu//stock/181; RRID: CVCL_Z992) and Kc167 (DGRC Stock 1; https://dgrc.bio.indiana.edu//stock/1; RRID: CVCL_Z834) cells were acquired directly from the *Drosophila* Genomics Resource Center (DGRC) (Schneider 1972; Cherbas *et al.* 1988). Both cell lines were cultured in Schneider’s *Drosophila* Medium supplemented with 10% fetal bovine serum and 1× penicillin–streptomycin–glutamine (Gibco). RNA from S2-DRSC cells was used for reverse transcription with Superscript III (Invitrogen) to make cDNA. This cDNA and Apex Taq Red (Genesee Sci) were used with T7 promoter-fusion primers (Supplementary Table 1) to amplify *B52*. Sequences for T7-fusion primers were derived from cell-screening “R” and “S” primer sets from the *Drosophila* RNAi Screening Center (DRSC) (Hu *et al.* 2020). PCR products served as templates for the overnight *in vitro* transcription with MEGAscript (Invitrogen) to generate double-stranded RNA for RNA interference (RNAi). Approximately 15 µg of dsRNA was added to each sample in a separate well of a 6-well tissue culture dish. The dsRNA was added on the first, third, and fifth days of the treatment. Samples were taken for RNA isolation on day 6.

HeLa and HepG2 cell pellets were generously supplied by the labs of K.A. Fields and B.T. Spear, respectively. Human cell pellets were immediately put in TRIzol reagent (Invitrogen) and RNA isolated for RT-PCR with human *RPS21e*-specific primers, as indicated below.

### Western blotting

Protein was isolated from wild-type and sorted larvae approximately 4 days post-egg laying. Protein was isolated by crushing larvae in 1× RIPA Buffer (Thermo Scientific) supplemented with Halt Protease (Thermo Scientific) and Halt Phosphatase (Thermo Scientific) inhibitor cocktails. Lysates were precipitated with trichloroacetic acid, washed with methanol, and dried (80°C for 10 min) before resuspension in 1× Sample Loading Buffer (*LI-COR*). Protein samples were separated on NuPAGE 4–12% Bis–Tris gels in 1× NuPAGE MES SDS running buffer. Separated proteins were transferred to 0.2-µm low fluorescence PVDF (Thermo Scientific) and blocked with Odyssey Blocking Buffer (TBS) (*LI-COR*). A rabbit polyclonal antibody was generated to an antigen for the protein product of *CG33057* by ABclonal. A *CG33057* gene fragment (amino acids 1–212) was subcloned into the pGEX-4T-AB1 vector for protein expression and purification. Primary antibodies for CG33057, RpS21 (Abcam # ab90874), and RpS6 (C.896.4) (Invitrogen) were used here at a 1:2,000 concentration. The monoclonal antibody to α-tubulin (4A1-s) developed by M.T. Fuller at Stanford University was obtained from the Developmental Studies Hybridoma Bank, created by the NICHD of the NIH and maintained at The University of Iowa, Department of Biology, Iowa City, IA, USA. The α-tubulin antibody was used at a 1:30,000 concentration. *LI-COR* infrared IR dye-conjugated anti-mouse and -rabbit secondaries were used at a 1:15,000 concentration to image and quantify proteins with an Odyssey Imager (*LI-COR*) and Image Studio Lite software (*LI-COR*). For statistical analysis, *P*-values were determined with a Student’s *t*-test.

### RNA isolation, RT-PCR, and real-time PCR

RNA was isolated form crushed larvae approximately 4 days post-egg laying. RNA was isolated with TRIzol reagent according to the manufacturer’s protocol. Isolated RNA was subjected to an additional round of DNase with TURBO DNase (Invitrogen), followed by phenol chloroform extraction. Reverse transcription (RT) was performed with Superscript III (Invitrogen) and random hexamers. PCR was performed with Apex Taq Red (Genesee Sci), and limited cycles of amplification with intron flanking oligos. PCR products were separated on an agarose gel in 1× TBE, stained with GelRed (Biotium), and imaged on a Gel Doc Ez (BIO-RAD). Separated bands were quantified on unaltered images with Image Lab 6.0.1 software (BIO-RAD). PSI was quantified as indicated above, as the percentage of spliced over total transcripts. For statistical analysis, *P*-values were determined with a Student’s *t*-test. Real-time PCRs of cDNA were conducted on a StepOnePlus System (Applied Biosystems), using Maxima (Thermo Scientific) or PowerUp (Applied Biosystems) SYBR Green/ROX master mixes. Three biological replicates were tested for each genotype. The ΔΔCt method was used to quantify differences, and *P*-values were determined with a Student’s *t*-test. Gene-specific primer sequences are listed in Supplementary Table 1.

### RNA-seq analyses

RNA-seq analysis was performed on original fastq files that were previously deposited in the NCBI Gene Expression Omnibus (GEO) (Edgar *et al.* 2002). Files are indicated below. Transcript abundance was quantified with kallisto (Bray *et al.* 2016) and differential analysis was performed with sleuth (Pimentel *et al.* 2017). HISAT2 (Kim *et al.* 2019) and Samtools (Danecek *et al.* 2021) were used to align RNA-seq reads to Release 6 of the *Drosophila melanogaster* genome (Hoskins *et al.* 2015) for visualization and Sashimi plot comparison with the Integrative Genomics Viewer (Robinson *et al.* 2011, 2017).

## Results

### RNA-seq uncovered an increase in *RpS21* intron 3 retention in fly *Phax^SH/SH^* mutants

**Fig. 1.**
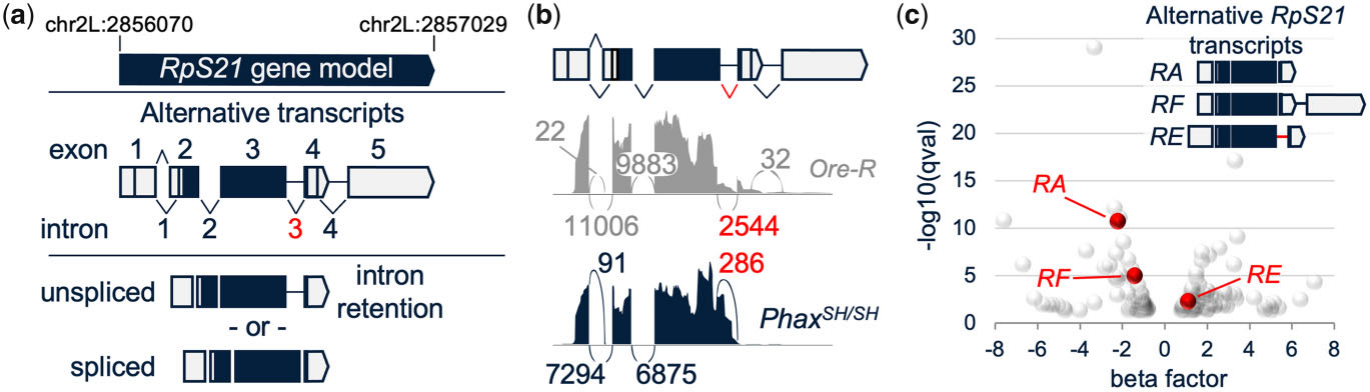
RNA-seq evidence for the alternative splicing of *RpS21* transcripts in *Phax^SH/SH^* mutants. a) *RpS21* gene model that includes constitutive and alternative splicing patterns of *RpS21*. Top: *Drosophila RpS21* gene model on chromosome 2L (chr2L); middle: alternative *RpS21* transcripts with numbered exons and introns. Splicing across intron 1 includes alternative 3′-splice sites. Splicing across intron 2 is constitutive. Introns 3 and 4 are alternatively spliced or retained. The right-side angled points of the boxes for exons 4 and 5 indicate sites of alternative polyadenylation. Exon 1 includes alternative transcription start sites. Bottom: transcripts with the conserved intron 3 retention (unspliced) or alternatively spliced exon 3 to exon 4 (spliced). Note, the vast majority of *RpS21* transcripts in the whole animals and the cell lines tested here utilize the proximal polyadenylation signal in exon 4. b) A sashimi plot graph of exon junction reads derived from RNA-seq of wild-type *Oregon-R* (*Ore-R*) and *Phax^SH/SH^* mutant (*Phax^SH/SH^*) animals. Curved lines indicate the locations of junction reads that span the relative locations indicated in the alternative *RpS21* transcript model at the top. The numbers indicate the total number of reads spanning those specific exon junctions. Reads spanning the exon 3 to exon 4 junction are indicated in red. c) Volcano plot of transcript differences between wild-type *Oregon-R* and *Phax^SH/SH^* mutant animals. Transcripts with higher levels in the *Phax^SH/SH^* mutants are on the right, and transcripts with lower levels are on the left. The *y*-axis is a negative log base 10 of the *q*-value, which is a false discovery rate (FDR) adjusted *p*-value (FDR < 0.05). The *x*-axis is a beta factor, an approximation of a normalized fold change, using the Wald test of the sleuth R package. Alternative differentially expressed *RpS21* transcripts are indicated in red.

Prior transcriptome analysis uncovered an overlapping set of alternative splicing changes in *Drosophila* snRNP biogenesis mutants. Notably, *Phax* and *Smn* mutants exhibited a large change in alternative splicing across intron 3 of the *Ribosomal protein S21* (*RpS21*) gene (Garcia *et al.* 2016). As shown here in RNA-seq data from *Phax^SH/SH^* mutants, a disruption of snRNA nuclear export and snRNP biogenesis leads to a large increase in intron 3 retention in *RpS21* transcripts, and a parallel decrease in reads spanning the *RpS21* exon 3 and 4 splice junction surrounding intron 3 (Fig. 1, a and b) (Garcia *et al.* 2016). These changes were uncovered previously in global alternative splicing analysis (Garcia *et al.* 2016) and in transcript-specific analysis here (Fig. 1c and Supplementary Table 2). *Phax^SH/SH^* mutants display an increase in transcripts for the *RpS21-RE* isoform that retains intron 3, and a corresponding decrease in *RpS21-RA* and *-RF* isoforms, which splice out intron 3 (Fig. 1c). The phenotypic consequences of these changes in *RpS21* intron retention are unknown.

### The splicing pattern of the predominant terminal exon of *RpS21* is highly conserved from flies to humans

**Fig. 2.**
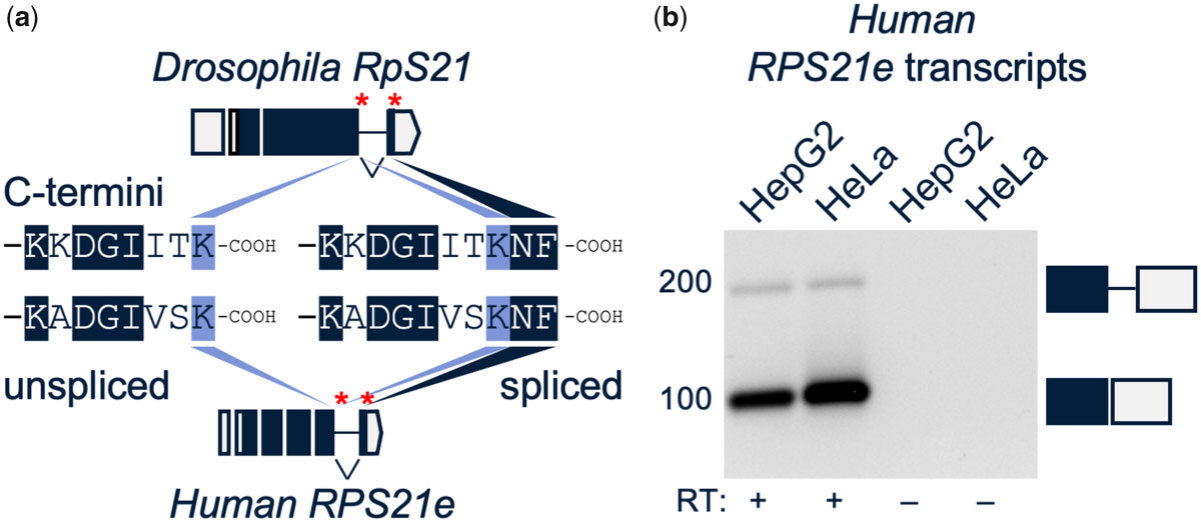
Conserved features of the terminal *RpS21* splice junction that overlaps the protein coding sequences. a) Transcript models of fly *RpS21* top and human *RPS21e* bottom. Alternative stop codons are indicated with asterisks. Middle: alternative carboxy-terminal (C-termini) ends encoded by unspliced and spliced transcripts. Identical amino acids are shaded, and the conserved terminal lysine (K) is shaded separately. b) Intron retention of human *RPS21e* transcripts. Image of a GelRed-stained agarose gel of RT-PCR products, amplified with primers that flank the terminal exon junction of human *RPS21e* transcripts. Image was converted to grayscale, inverted, and contrast adjusted. Right: boxes depict the mobility of PCR products corresponding to unspliced (top) and spliced (bottom) transcripts.

Splicing of the third intron of *RpS21* pre-mRNA modulates the carboxy-terminus of the encoded RpS21 protein (Fig. 2a). The last 3 amino acids at the carboxy-terminus of the RpS21 protein are lysine (K), asparagine (N), and phenylalanine (F). The splice junction between exons 3 and 4 spans the K codon. The 3 carboxy-terminal amino acids of fly RpS21 are conserved with the human RPS21e protein, and, most notably, the human *RPS21e* transcripts also include a splice junction that spans the terminal K codon (Fig. 2a). In unspliced *RpS21* transcripts, the intron contains the third wobble base of the terminal K codon and a following stop codon. The same pattern is conserved in the corresponding intron of human *RPS21e* transcripts. Human cell lines also show evidence of retention of the corresponding intron of *RPS21e* (Fig. 2b). Hence, this is an exceptionally well-conserved splicing pattern from flies to humans.

### *Phax^SH/SH^* mutants exhibit an expected decline in viability but a surprising lack of any adult escapers

As this conserved splicing event in *RpS21* transcripts was altered in *Phax^SH/SH^* mutant flies, *Phax^SH/SH^* mutants and a *UAS*-*Phax* rescue transgene were used to determine how changes in this splicing event might contribute to organismal viability. In agreement with our previous findings (Garcia *et al.* 2016), the *Phax^SH/SH^* mutants exhibited a large decrease in viability, as compared to wild-type *Oregon-R* controls (Supplementary Fig. 1a). Also, as before (Garcia *et al.* 2016), this decrease in viability was partially rescued by the ubiquitous expression of a wild-type *UAS*-*Phax* transgene with an *armadillo*-*GAL4* driver (Supplementary Fig. 1a). However, unlike the previous study, ubiquitous expression of the *UAS*-*Phax* transgene did not lead to the eclosure of any adult flies (Supplementary Fig. 1a). In our previous study, approximately 30% of *Phax*-transgene expressing larvae developed to adulthood (Garcia *et al.* 2016). As the yeast-derived *GAL4*-*UAS* system is optimal at approximately 27°C, we also assayed viability at 27°C. Temperature did not account for the discrepancies between our current and prior viability studies, as viability at 27°C was roughly equivalent to the viability at 23°C (Supplementary Fig. 1, a and b). Prior to our rescue experiments and the site-specific integration of the *UAS*-*Phax* transgene into the *Phax^SH/SH^* mutant background, adult escapers, homozygous for the P element insertion in the *Phax* gene, were intermittently detectable in different stocks of this *Phax^SH/SH^* mutant line (Oh *et al.* 2003; Kahsai *et al.* 2016; Garcia *et al.* 2016). We hypothesized that subtle genetic background differences might contribute to the observed differences in the viability of different stocks of the *Phax^SH/SH^* mutant and in our transgenic rescue experiments.

### Proper splicing across intron 3 of *RpS21* is linked to *Phax* expression

**Fig. 3.**
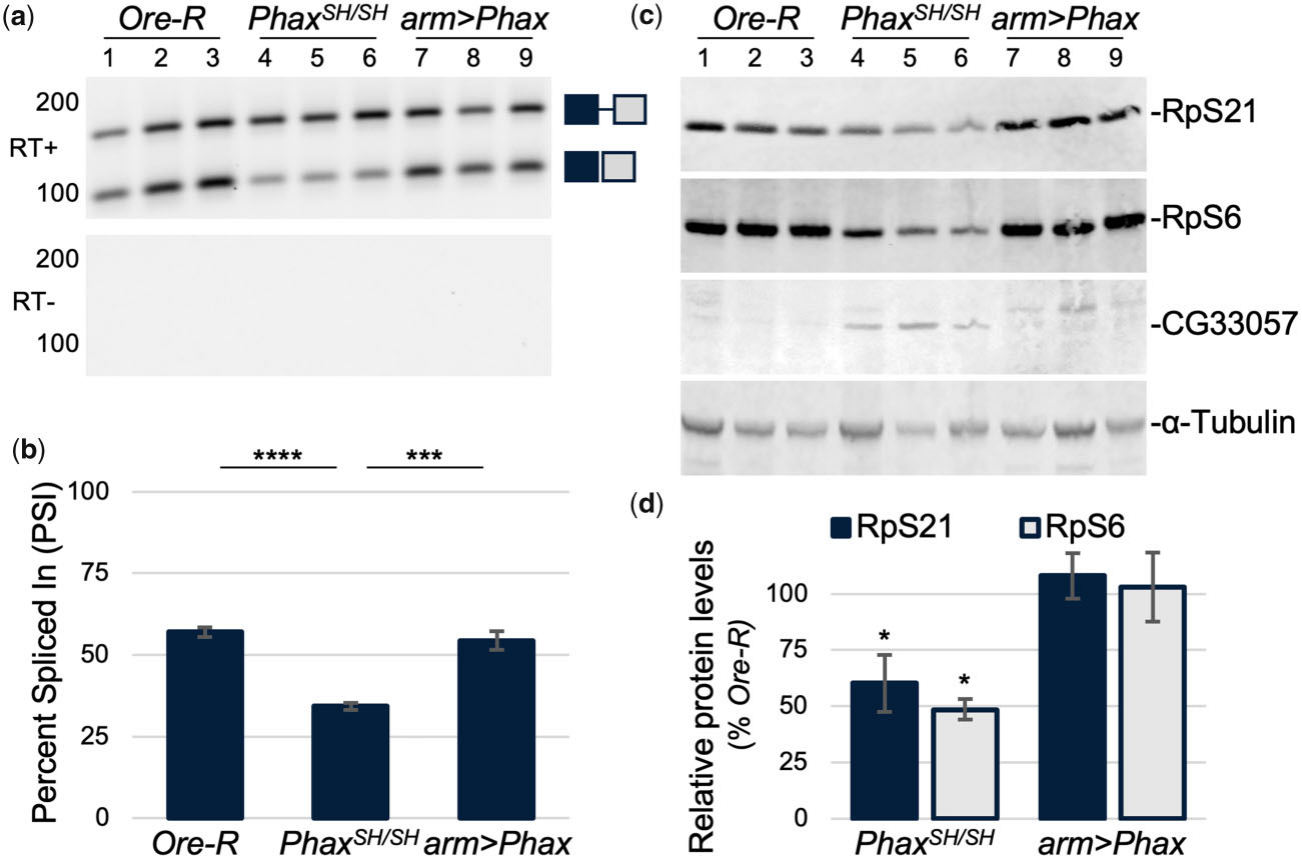
Differential abundance of *RpS21* transcripts and RpS21 protein levels in *Phax^SH/SH^* mutant and rescue animals. a) Increase in *RpS21* intron 3 retention in *Phax^SH/SH^* mutants and corresponding decrease upon expression of the Phax transgene. Transgenic rescue animals (*arm>Phax*) utilized an *armadillo-GAL4* driver (*arm*) and a *UAS:Phax-mVenus* transgene (*Phax*) in the *Phax^SH/SH^* mutant background. Image of a GelRed-stained agarose gel of RT-PCR products, amplified with primers flanking the predominant terminal exon junction, encompassing intron 3 of *Drosophila RpS21* transcripts. Gels include PCR products or negative controls from reactions with (RT+) or without reverse transcriptase (RT−), respectively. Gel image was adjusted as in Fig. 2b. Of note, *RpS21* terminal intron 4 from Fig. 1a is rarely spliced out of *RpS21* transcripts that utilize the distal polyadenylation signal. *RpS21* distal polyadenylated transcripts are also relatively low compared to proximal polyadenylated transcripts in the whole animals and cells used here. b) Quantification of the percentage of splicing from exon 3 to exon 4 in (a). The graph is the percentage of spliced to total transcripts, shown as PSI. c) Western blots for the indicated proteins. d) Quantification of the RpS21 and RpS6 protein levels relative to α-tubulin from (c). Relative levels of these proteins in *Oregon-R* were set to 100%. *P < 0.05; ***P < 0.001; and ****P < 0.0001.

Changes in *RpS21* intron 3 retention parallel the observed changes in viability of *Phax^SH/SH^* mutant and rescue animals. The splicing across *RpS21* intron 3 is normally inefficient, as wild-type *Oregon-R* larvae exhibit a high baseline level of intron 3 retention (Fig. 3a). In *Oregon-R* animals, the percentage of fully spliced *RpS21* transcripts across intron 3 averaged a low 56 PSI (Fig. 3b). Splicing across intron 3 was worse in the *Phax^SH/SH^* mutants, evident as a visible increase in the ratio of unspliced to spliced transcripts (Fig. 3a) and a decrease in the percentage of fully spliced transcripts to 34 PSI (Fig. 3b). These changes in *RpS21* splicing were fully rescued by ubiquitous expression of the *UAS-Phax* transgene (Fig. 3, a and b). Nevertheless, the rescue of *RpS21* alternative splicing did not fully restore the developmental progression in *UAS-Phax* expressing animals.

### *Phax^SH/SH^* mutant changes in alternative splicing affect the steady-state levels of encoded proteins

Anticipated changes in steady-state protein levels accompanied the changes in alternative splicing in the *Phax^SH/SH^* mutants. *Phax^SH/SH^* mutants exhibited a decrease in steady-state levels of RpS21 protein relative to a tubulin loading control (Fig. 3, c and d). Levels of RpS21 protein were rescued by expression of the *UAS-Phax* transgene (Fig. 3, c and d). Protein levels of another small subunit protein, ribosomal protein S6 (RpS6), were also down in the *Phax^SH/SH^* mutants (Fig. 3, c and d). Although small changes in *RpS6* transcripts were found in our transcript-specific analysis here, changes in *RpS6* alternative splicing were not uncovered in our more stringent prior analysis (Garcia *et al.* 2016). Hence, changes in RpS6 protein levels could reflect the more dramatic changes in *RpS21* alternative splicing or broader splicing disruptions exhibited by the *Phax^SH/SH^* mutants. As an additional control for anticipated protein level changes, we blotted for the protein product of the computed gene *CG33057*, which encodes an ortholog of the yeast tRNA 2′-phosphotransferase protein (TPT1). Transcripts for *CG33057* are contained entirely within an intron of transcripts for the *monkey king protein* (*mkg-p*) gene that is retained in snRNP biogenesis mutants (Garcia *et al.* 2016). We detected an increase in levels of a protein of the expected molecular weight for the fly TPT1 ortholog in lysates from *Phax^SH/SH^* mutants (Fig. 3c). Protein levels of the putative fly TPT1 ortholog returned to baseline levels upon expression of the *UAS-Phax* transgene (Fig. 3c). However, like the noted changes in *RpS21* alternative splicing, rescue of protein levels was not sufficient to restore the developmental progression of *UAS-Phax* transgene expressing animals.

### Common *Drosophila* flies and cell lines have different alleles of the *RpS21* gene

**Fig. 4.**
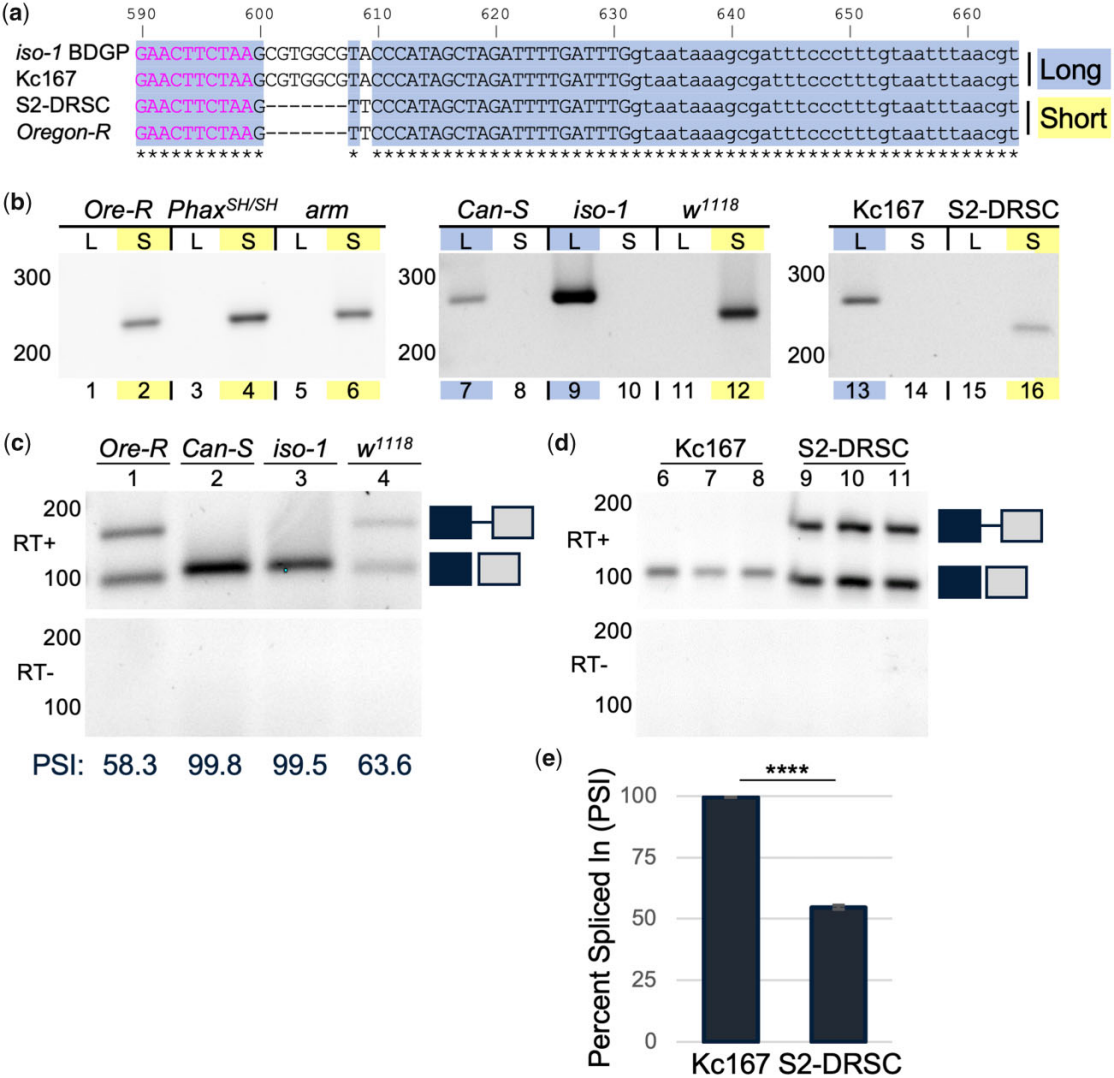
Alternate *RpS21* alleles and corresponding differences in transcript abundance. a) Multiple sequence alignment of the predominant exon 4 of *RpS21*. Subcloned and sequenced DNA from Kc167 and S2-DRSC cell lines and *Oregon-R* flies was aligned to the *Drosophila* genome reference strain (*iso-1*). Identical bases are shaded in blue with an asterisk below. C-terminal coding sequences are indicated on the left in pink. Descriptors for the long allele (L) top and short allele (S) bottom are also shaded to coincide with the genotyping in (b), where allele-specific primers were used to amplify DNA from the indicated flies and cells. GelRed-stained agarose gels of PCR products were adjusted as in Fig. 2b. c) Relative abundance of *RpS21* transcripts in the indicated fly lines. Inverted GelRed-stained agarose gels of RT-PCR products, as indicated previously. Quantification of the blot in PSI is indicated below each lane. d) Relative abundance of *RpS21* transcripts in Kc167 and S2-DRSC cells. Inverted GelRed-stained agarose gels of RT-PCR products, as indicated previously. e) Quantification of the PSI for transcripts from (d). ****P < 0.0001.

DNA sequencing revealed a surprising allelic heterogeneity in exon 4 of *RpS21*, immediately downstream of intron 3, in common laboratory flies and cell lines. Using *RpS21* gene-flanking primers, we subcloned and sequenced the *RpS21* gene in *Oregon-R* flies, S2-DRSC cells, and Kc167 cells. Surprisingly, *RpS21* exon 4 from S2-DRSC cells and *Oregon-R* flies contained a 7-nucleotide deletion and an additional adenine to thymine transversion relative to Kc167 cells and the *Drosophila* genome reference strain *iso-1* (Fig. 4a). Available genome assemblies also support the identified allelic heterogeneity in *RpS21* exon 4 amongst different *Drosophila* cell lines (Supplementary Fig. 2). This subtle genetic difference was used to design allele-specific primers to genotype additional fly lines. The *Phax^SH/SH^* mutants, *Armadillo*-*GAL4* rescue line (*arm*), and the widely used *w^1118^* mutant contained the short (S) *RpS21^S^* allele found in *Oregon-R* flies and S2-DRSC cells (Fig. 4b). The alternative wild-type *Canton-S* flies had the long (L) *RpS21^L^* allele like *iso-1* flies and Kc167 cells (Fig. 4b). The genetic difference in exon 4 overlapped a predicted Exonic Splicing Enhancer (ESE) in the longer *RpS21^L^* allele (Cartegni *et al.* 2003). Therefore, we hypothesized that the discrete genetic differences in exon 4 could affect the splicing across intron 3 of *RpS21* pre-mRNA.

### Allelic differences in the *RpS21* gene correspond to differences in the splicing of *RpS21* transcripts

Indeed, the genetic differences in exon 4 correlated with the expected differences in intron 3 retention in *RpS21* transcripts (Fig. 4, c and d). *RpS21* transcripts from *Oregon-R* and *w^1118^* flies exhibited a high level of intron 3 retention and a relatively low, approximately 60 PSI, level of spliced transcripts. In contrast, *Canton-S* and *iso-1* flies with the longer *RpS21* allele, containing the predicted ESE, had little to no intron 3 retention and a high, greater than 99 PSI, level of spliced transcripts (Fig. 4c). This pattern was also evident in *Drosophila* Kc167 cells with the long *RpS21* allele *vs.* S2-DRSC cells, lacking the putative ESE (Fig. 4, d and e). Kc167 cells exhibited greater than 99 PSI with no *RpS21* intron 3 retention, and S2-DRSC cells had a high level of intron 3 retention and a low, 55 PSI, level of spliced transcripts (Fig. 4, d and e). The increased splicing efficiency of *RpS21* transcripts from the longer allele supports the prediction that the additional GC-rich sequences in exon 4 function as a strong ESE.

### Predicted ESE sequences in exon 4 of *RpS21* transcripts enhance the splicing of exon 3 to exon 4

We constructed an *RpS21* mini-gene splicing reporter to further test the prediction that the additional sequences in exon 4 of the long *RpS21* allele function as an ESE. The minimal *RpS21* mini-gene spanned the 3′-end of exon 3 to the end of exon 4 with (*miniS21^L^*) and without (*miniS21^S^*) the putative ESE in exon 4 (Supplementary Fig. 3a). The splicing reporter utilized a heterologous *Actin5C* promoter and an SV40 polyadenylation signal (Supplementary Fig. 3a). We created an additional mini-gene without the predicted ESE sequences that contains a consensus 5′-splice site (*miniS21^S^* A>G) (Supplementary Fig. 3a). The A>G mutation was designed to test the potential contribution of the unpaired A nucleotide in the 5′-spliced site to *RpS21* intron 3 retention. As expected, the PSI of the *miniS21^S^* A>G reporter was significantly higher than the *miniS21^S^* reporter with the endogenous 5′-splice site, supporting a small contribution of this seemingly weak 5′-splice site to *RpS21* intron 3 retention. However, the splicing efficiency of all 3 mini-gene reporters was greater than 80 PSI, which exceeded the inefficient splicing of endogenous transcripts from the *RpS21* short allele (Supplementary Fig. 3, b and c). This suggests that additional sequences that were not included in the mini-gene splicing reporter likely contribute to the high level of *RpS21* intron 3 retention in transcripts encoded by the short allele. Nevertheless, the *RpS21* mini-gene with the putative ESE in exon 4 (*miniS21^L^*) produced transcripts with significantly less intron retention and a greater PSI than transcripts from either mini-gene lacking the ESE sequences, which further supports the hypothesis that the additional nucleotides in the long allele function to enhance the splicing of *RpS21* exon 3 to exon 4 (Supplementary Fig. 3, b and c).

### Knockdown of the B52 splicing factor decreases *RpS21* intron 3 retention in *RpS21* transcripts without the predicted ESE in exon 4

**Fig. 5.**
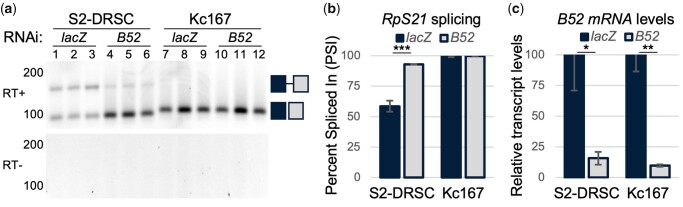
RpS21 alternative splicing upon control or B52 RNAi depletion. a) RT-PCR of *RpS21* in control (*lacZ*) and B52 depleted S2-DRSC and Kc167 cells. Images were inverted and contrast adjusted as indicated previously. b) Quantification of PSI of *RpS21* transcripts from (a). c) Real-time qRT-PCR of *B52* transcript levels relative to *α-tubulin* transcripts, determined by the ΔΔCt method. *P < 0.05; **P < 0.01; and ***P < 0.001.

A prior cross-comparison of published RNA-seq data for local splicing variations revealed a decrease in *RpS21* intron 3 retention upon RNAi knockdown of mRNA for the splicing factor B52 in *Drosophila* S2 cells (Srivastava *et al.* 2021). As expected from these transcriptomic findings, *B52* knockdown in S2-DRSC cells here led to a decrease in *RpS21* intron 3 retention and a corresponding 30% increase in the percentage of spliced *RpS21* transcripts (Fig. 5, a–c). However, *B52* knockdown in Kc167 cells did not appear to affect the splicing across intron 3 of *RpS21* in those cells, as the splicing efficiency of *RpS21* in Kc167 cells without B52 knockdown is already very high (>99 PSI) (Fig. 5, a–c). In other words, the long allele of *RpS21*, with the predicted ESE in exon 4, appears to short circuit or counter the inhibitory regulation of pre-mRNA splicing of *RpS21* exon 3 to exon 4 by B52.

### The *RpS21* ESE-containing allele suppresses the pupal lethality of the *Phax^SH/SH^* mutants

**Fig. 6.**
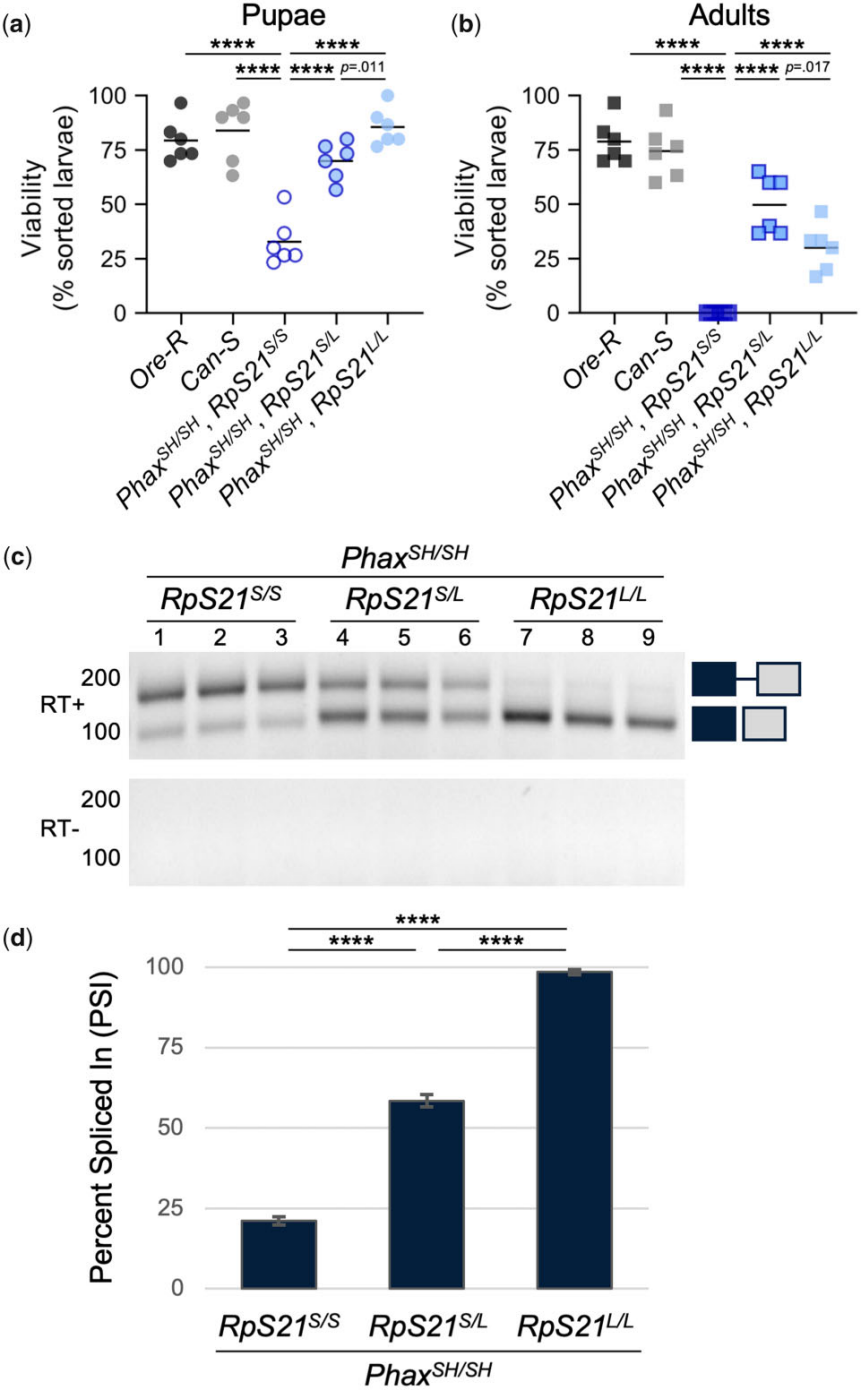
Inverse correlation of *RpS21* intron 3 retention with the viability of *Phax^SH/SH^* mutants. Pupae (a) and adults (b) quantified as a percentage of sorted larvae from the genotypes indicated on the *x*-axis. Horizontal lines indicate the mean for each genotype. ****P < 0.0001. c) RT-PCR of *RpS21* from the indicated genotypes. Images were inverted and contrast adjusted as indicated previously. d) Quantification of PSI of *RpS21* transcripts from (c). ****P < 0.0001. Relative *RpS21* intron 3 retention and percentage of spliced *RpS21* transcripts in *Phax^SH/SH^* mutants with the different *RpS21* alleles.

With mounting evidence for the high splicing efficiency of the long *RpS21* allele, we asked whether this allele could restore the developmental progression of snRNP biogenesis mutants. Notably, the long allele of *RpS21* was able to partially restore the developmental progression of *Phax^SH/SH^* mutants (Fig. 6, a and b). At the pupal stage, the presence of the long *RpS21* allele (*RpS21^L^*) significantly improved the viability of *Phax^SH/SH^* mutants in an allele dose dependent manner (Fig. 6a). Furthermore, unlike *Phax^SH/SH^* mutants with only the short *RpS21* allele (*RpS21^S/S^*), *Phax^SH/SH^* mutants that were either heterozygous for *RpS21* (*RpS21^S/L^*) or homozygous for the long allele (*RpS21^L/L^*) produced adult escapers (Fig. 6b). To date, no progenies have been observed from any of the adult *Phax* escapers. Nevertheless, we conclude that different *RpS21* alleles likely account for our prior observations of intermittent adult escapers in *Phax^SH/SH^* mutant stocks.

### The *RpS21* ESE-containing allele decreases *RpS21* intron 3 retention in the *Phax^SH/SH^* mutants in an allele dose dependent manner

In the *Phax^SH/SH^* mutant background, the presence of the long *RpS21^L^* allele rescues the splicing of exon 3 to exon 4 of *RpS21* transcripts (Fig. 6c). *Phax^SH/SH^* mutant larvae that are homozygous for the short *RpS21^S^* allele (*RpS21^S/S^*) exhibited the highest level of intron 3 retention and the lowest level of exon 3 and 4 splicing (21 PSI) relative to *Oregon-R* controls (Fig. 6, c and d) or larvae with even a single copy of the long *RpS21^L^* allele (Fig. 6, c and d). *Phax^SH/SH^* mutants, which are heterozygous for the *RpS21* short and long alleles (*RpS21^S/L^*), exhibited similar levels of intron 3 retention and splicing (58 PSI) as wild-type *Oregon-R* animals that are homozygous for the short allele (*RpS21^S/S^*) (Figs. 6, c and d and 3, a and b). *Phax^SH/SH^* mutants that are homozygous for the long *RpS21^L^* allele (*RpS21^L/L^*) have levels of intron 3 retention and splicing (98 PSI) that are like wild-type *Canton-S* animals (99 PSI) with no genetic perturbations of snRNP biogenesis (Fig. 6, c and d). Hence, we conclude that the long *RpS21^L^* allele is sufficient to rescue the perturbation of *RpS21* splicing caused by P element mutations in the *Phax* gene.

## Discussion

The alternative splicing of pre-mRNA for RPs has the potential to regulate the overall abundance and composition of ribosomes. Changes in either ribosome abundance or composition can cause specific changes to gene expression and affect the protein synthesis of select transcripts (Horos *et al.* 2012; Khajuria *et al.* 2018). Our prior transcriptome analysis of snRNP biogenesis mutants uncovered specific changes to the alternative splicing of *RP* pre-mRNA (Garcia *et al.* 2016). As shown here, the *Phax^SH/SH^* mutant changes to *RpS21* pre-mRNA correlated with a decrease in RpS21 and RpS6 protein abundance that is consistent with a putative decrease in ribosome numbers. In addition, *RpS21* intron 3 retention encodes an RpS21 protein isoform that is 2 amino acids shorter at the carboxy-terminus. Thus, in addition to ribosome abundance, the modulation of the alternative splicing of *RP* pre-mRNA can subtly alter ribosome composition.

Disruptions to ribosome numbers have been previously linked to disease. Mutations in numerous *RP* coding genes, other than human *RPS21*e, are associated with the blood disorder Diamond-Blackfan anemia (DBA) (Narla and Ebert 2010; Horos *et al.* 2012; Mills and Green 2017; Khajuria *et al.* 2018; Aspesi and Ellis 2019; Costa *et al.* 2020). DBA-associated *RP* gene mutations have been linked to reductions in ribosome levels (Khajuria *et al.* 2018). A similar decrease in ribosome numbers has been observed in mouse models of the neuromuscular disease Spinal Muscular Atrophy (SMA) (Bernabò *et al.* 2017). SMA is caused by mutations in the human *Survival Motor Neuron 1* (*SMN1*) gene (Lefebvre *et al.* 1995). In addition to a reduction in axonal ribosomes, SMA model mice exhibited lower levels of Rps6 protein and a wide-spread decrease in the translation efficiency of *RP* transcripts, including transcripts for mouse *Rps21* and *Rps6* (Bernabò *et al.* 2017). The reductions of fly RpS21 and RpS6 proteins in the *Phax^SH/SH^* mutants are consistent with the finding of disrupted ribosome homeostasis in SMA model mice. How specific alternative splicing events contribute to RP levels and overall ribosome numbers remains to be determined, but findings here suggest that mis-splicing of *RP* coding transcripts may contribute to the observed reduction in RP levels and possibly ribosome numbers. Importantly, however, the long ESE-containing *RpS21* allele has, to date, been insufficient to suppress the larval lethality of fly *Smn* null mutants.

In the absence of the strong ESE in exon 4, *RpS21* alternative splicing was negatively regulated in *trans* by the SR-rich B52 splicing factor. The human ESEfinder predicts a Serine and Arginine Rich Splicing Factor 6 (SRSF6)-binding site, nucleotides –GGCGUA–, that overlaps the allelic difference in exon 4 of *RpS21* transcripts from the longer *RpS21^L^* allele (Fig. 4a) (Cartegni *et al.* 2003). To date, our RNAi screens have yet to identify an orthologous *Drosophila* splicing factor and/or component of the 3′-cleavage and polyadenylation machinery that binds to this putative ESE in exon 4 to enhance *RpS21* splicing. The negative regulation of *RpS21* splicing by B52, occurring in *RpS21* transcripts without this ESE, likely requires additional *cis*-regulatory sequences beyond intron 3 and proximal sequences in the adjacent exons. The minimal *RpS21* splicing reporter without the ESE had a notably high PSI (>80 PSI) relative to the low PSI (∼58 PSI) of corresponding endogenous *RpS21* transcripts. In addition to a heterologous promoter and polyadenylation signal, the minimal reporter included only intron 3, the short downstream exon 4, and a mere 100 out of 192 nucleotides of upstream exon 3. B52 has a demonstrated affinity for structured RNA with accessible single-stranded binding sites (Shi *et al.* 1997). Binding of B52 to *RpS21* transcripts may therefore require additional upstream or downstream sequences necessary for proper RNA structure.

Overexpression of B52 has been linked to increased growth and depletion to decreased growth (Fernando *et al.* 2015; Wada *et al.* 2021). In addition to *RpS21*, third-party analysis of published RNA-seq data of B52 knockdowns in S2 cells uncovered alternative splicing of *yorkie* transcripts, a transcriptional co-activator in Hippo signaling (Srivastava *et al.* 2021). Hippo signaling is a highly conserved pathway that controls organ size (Halder and Johnson 2011; Hariharan 2015; Yu *et al.* 2015; Meng *et al.* 2016). Depletion of 1 copy of the *yorkie* gene enhanced the dysregulated cell-growth phenotype of heterozygous *RP* mutants (Wada *et al.* 2021). Through the concerted regulation of the alternative splicing of *RpS21* and *yorkie* transcripts, splicing factors like B52 can synchronize the ribosome with cell growth signals.

Proteomic and phospho-proteomic studies of the human RPS21e protein have identified posttranslational modifications at the carboxy-terminus of the protein that are likely impacted by the alternative splicing of the terminal exon of *RPS21e* transcripts (Choudhary *et al.* 2009; Mertins *et al.* 2013, 2016; Akimov *et al.* 2018). Overall, our findings are consistent with a model for the regulation of RpS21 through the alternative splicing of the terminal *RpS21* coding exon and corresponding modulation of the carboxy terminus of the protein. The allele-specific *RpS21* alternative splicing patterns uncovered here will aid the study of the alternative splicing control of RpS21 protein isoforms and their potential roles in ribosome biogenesis and homeostasis.

## Data availability

All *Drosophila* stocks and plasmids are available upon request. The author affirms that all data necessary for confirming the conclusions of the article are present within the article, figures, and tables. RNA-seq analysis was performed on original fastq files from the following GEO series accession numbers: *Oregon-R*, GSE49587 https://www.ncbi.nlm.nih.gov/geo/query/acc.cgi?acc=GSE49587 and *Phax^SH/SH^*, GSE81121 https://www.ncbi.nlm.nih.gov/geo/query/acc.cgi?acc=GSE81121.

Supplemental material is available at *G3* online.

## Supplementary Material

jkac195_Supplementary_Data
