# Supplementary material for: Allele-specific alternative splicing of Drosophila Ribosomal protein S21 suppresses a lethal mutation in the Phosphorylated adaptor for RNA export (Phax) gene
Source: G3 (Bethesda). 2022 Aug 3;12(9):jkac195. doi: 10.1093/g3journal/jkac195 (PMC9434302; doi:10.1093/g3journal/jkac195)
Supplement: jkac195_Supplementary_Data [file jkac195_supplementary_data.zip › Suppl/Supplemental_Figure_Legends_G3-2022-403548.docx]

**Supplemental Fig. S1.** Viability of *Phax^SH/SH^* mutant and transgenic rescue animals. Graphs of the percentage of pupae from wild-type (*Ore-R*), *Phax^SH/SH^* mutant, and rescue (*arm>Phax*) sorted larvae at 23°C (a) and 27°C (b). Transgenic rescue animals (*arm>Phax*) utilized an *armadillo*-*GAL4* driver (*arm*) and a *UAS:Phax*-*mVenus* transgene (*Phax*) in the *Phax^SH/SH^* mutant background. A Student’s *t* test was used to determine *p*-values: ** *p* < 0.01; *** *p* < 0.001; and **** *p* < 0.0001.

**Supplemental Fig. S2.** Manually curated multiple alignment of sequences from the modENCODE genomic assembly of *Oregon-R* and various fly cell lines. Representative sequences are shown relative to the reference strain *iso-1*. Aligned sequences span the start of exon 4 until just before the proximal polyadenylation signal. Aligned reads for the cell lines D20-C20 and ML-DmD20-c5 appeared to be heterozygous for the two *RpS21* sequences noted in the alignment. Asterisks at the bottom indicate identity.

**Supplemental Fig. S3.** *RpS21* alternative splicing mini-gene reporter. (a) Diagram of the *RpS21* splicing mini-gene reporter (*miniS21*) and sequences of the *RpS21* exon 3 5'-splice site (5'-SS) relative to consensus, complementary U1 snRNA, and mutant (A>G) sequences (Top). The reporter utilizes a *Drosophila* Actin5C (Act5C) promoter and a minimal SV40 polyadenylation signal (SV40pA). Portions of exons 3 and 4 are flanked with unique primer binding sites for selective PCR of cDNA from the reporter. IUPAC single letter nucleotide code indicates the consensus, pseudouridines (Ψ) are noted in the 5'-end of U1 snRNA, and the intronic GU adjacent to the splice-site is underlined. *RpS21* mini-gene reporter corresponding to the short RpS21 allele (*miniS21^S^*) and the long allele (*miniS21^L^*) containing the predicted ESE are indicated below. (b) RT-PCR of RNA from S2-DRSC cells transfected with the three versions of the *miniS21* reporter: *miniS21^S^*, a *miniS21^S^* with an A to G mutation in the 5'-splice site (*miniS21^S^* A>G), and the ESE containing *miniS21^L^*. Images were inverted, and contrast adjusted as indicated previously. (c) Quantification of (b). * *p* < 0.05 and ** *p* < 0.01.
