# Supplementary figures and images for: Allele-specific alternative splicing of Drosophila Ribosomal protein S21 suppresses a lethal mutation in the Phosphorylated adaptor for RNA export (Phax) gene
Source: G3 (Bethesda). 2022 Aug 3;12(9):jkac195. doi: 10.1093/g3journal/jkac195 (PMC9434302; doi:10.1093/g3journal/jkac195)

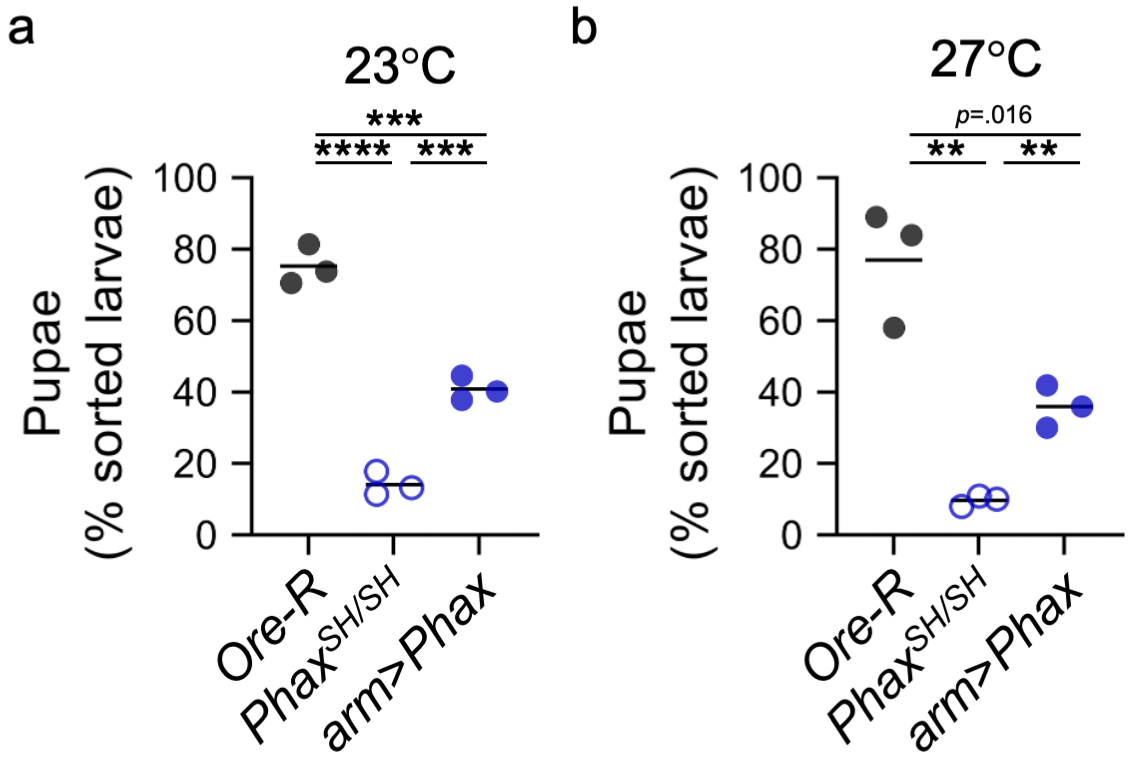

Supplement: jkac195_Supplementary_Data [file jkac195_supplementary_data.zip › Suppl/Supplemental_Figure_S1_G3-2022-403548.tif]

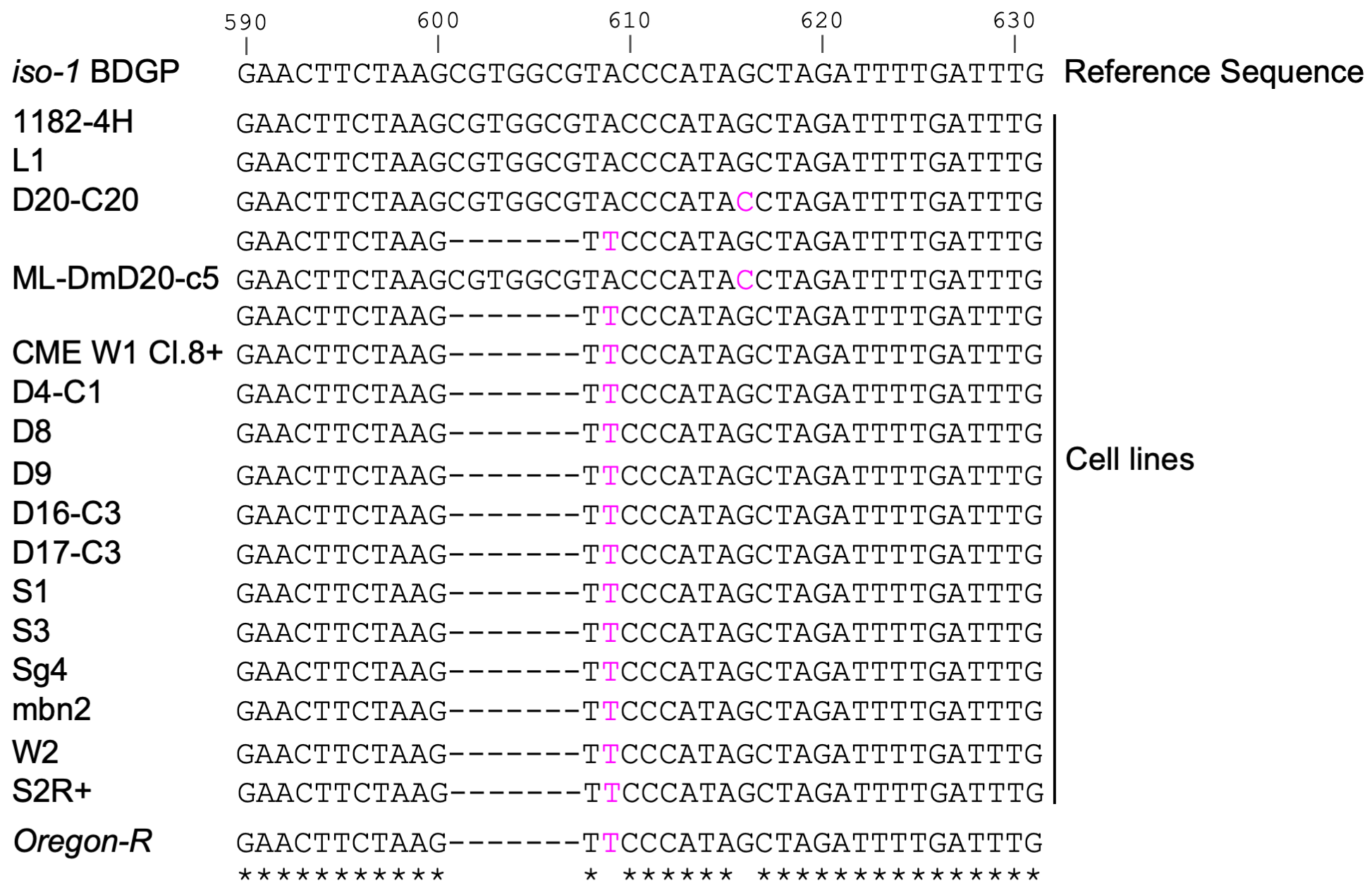

Supplement: jkac195_Supplementary_Data [file jkac195_supplementary_data.zip › Suppl/Supplemental_Figure_S2_G3-2022-403548.tif]

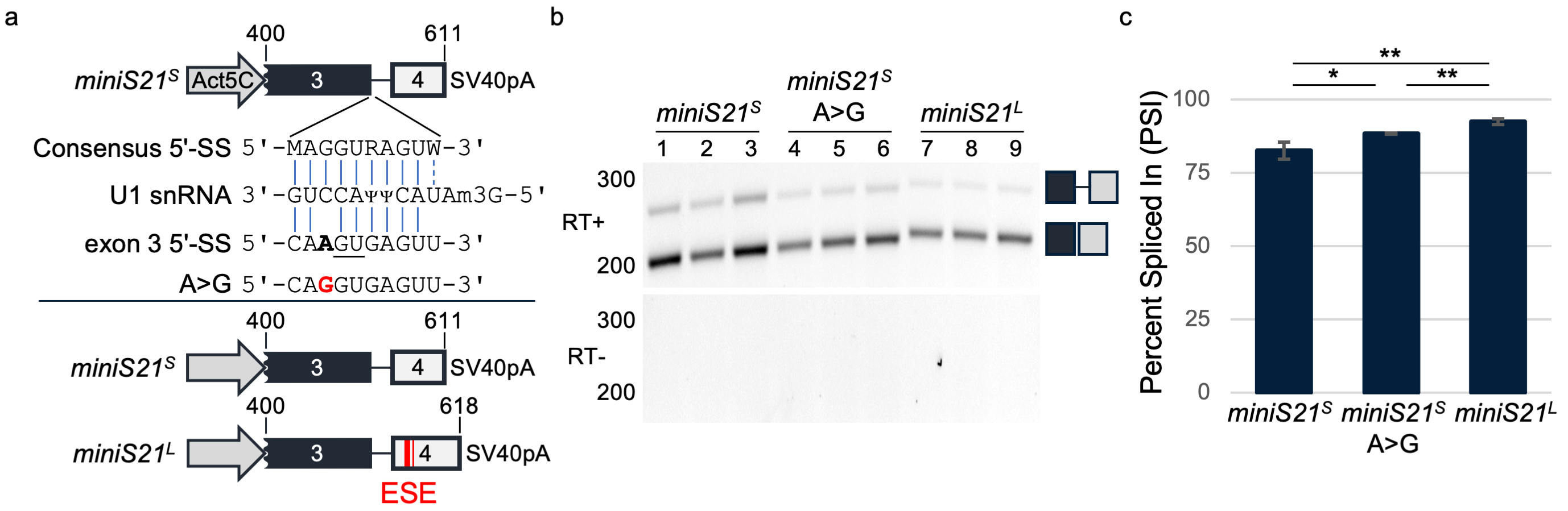

Supplement: jkac195_Supplementary_Data [file jkac195_supplementary_data.zip › Suppl/Supplemental_Figure_S3_G3-2022-403548.tif]
